# Testes and brain gene expression in precocious male and adult maturing Atlantic salmon (Salmo salar)

**DOI:** 10.1186/1471-2164-11-211

**Published:** 2010-03-30

**Authors:** Aoife Guiry, Denis Flynn, Sophie Hubert, Allan M O'Keeffe, Olivier LeProvost, Samantha L White, Patrick F Forde, Pamela Davoren, Benoit Houeix, Terry J Smith, Deirdre Cotter, Noel P Wilkins, Michael T Cairns

**Affiliations:** 1 National University of Ireland, Galway, University Road, Galway, Ireland; 2 Marine Institute, Furnace, Newport, Co Mayo, Ireland

## Abstract

**Background:**

The male Atlantic salmon generally matures in fresh water upon returning after one or several years at sea. Some fast-growing male parr develop an alternative life strategy where they sexually mature before migrating to the oceans. These so called 'precocious' parr or 'sneakers' can successfully fertilise adult female eggs and so perpetuate their line. We have used a custom-built cDNA microarray to investigate gene expression changes occurring in the salmon gonad and brain associated with precocious maturation. The microarray has been populated with genes selected specifically for involvement in sexual maturation (precocious and adult) and in the parr-smolt transformation.

**Results:**

Immature and mature parr collected from a hatchery-reared stock in January were significantly different in weight, length and condition factor. Changes in brain expression were small - never more than 2-fold on the microarray, and down-regulation of genes was much more pronounced than up-regulation. Significantly changing genes included isotocin, vasotocin, cathepsin D, anamorsin and apolipoprotein E. Much greater changes in expression were seen in the testes. Among those genes in the testis with the most significant changes in expression were anti-Mullerian hormone, collagen 1A, and zinc finger protein (Zic1), which were down-regulated in precocity and apolipoproteins E and C-1, lipoprotein lipase and anti-leukoproteinase precursor which were up-regulated in precocity. Expression changes of several genes were confirmed in individual fish by quantitative PCR and several genes (anti-Mullerian hormone, collagen 1A, beta-globin and guanine nucleotide binding protein (G protein) beta polypeptide 2-like 1 (GNB2L1) were also examined in adult maturing testes. Down-regulation of anti-Mullerian hormone was judged to be greater than 160-fold for precocious males and greater than 230-fold for November adult testes in comparison to July testes by this method. For anti-Mullerian hormone and guanine nucleotide binding protein beta polypeptide 2-like 1 expression changes in precocious males mirrored mature adults (November) but for collagen 1A and beta-globin the pattern was more complex.

**Conclusions:**

Expression changes in the fish brain during the process of precocious sexual maturation were small compared to those in the testes. Microarray analysis suggested down-regulation of housekeeping functions and up-regulation of a small number of specific processes. Transcriptional changes in the testes were much more pronounced with anti-Mullerian hormone playing a major role. Expression profiles for mature parr and maturing adult testes indicate subtle differences in gene expression between these two related groups.

## Background

Alternative reproduction tactics (ARTs) enable fish to exploit a wide spectrum of resources and to respond to conditions such as an imbalance of sexes in the population [1]. The opportunity for ARTs in fishes arises largely as a consequence of external fertilisation. Access to eggs for fertilisation can be by adult males or alternatively by precociously mature parr variously described as 'satellite males' or 'sneakers' [2,3]. The mature parr succeed mainly through speed or through being inconspicuous to adult males who might otherwise drive them off. The approach taken by an individual fish is believed to depend on the balance of energetic cost against reproductive payoff. The energetic cost of adult male reproduction in salmon is very high and few adults return to reproduce a second time [4,5]. However, the payoff is also high because an adult male can dominate an egg-laying female and almost all offspring are sired by that male. The energetic cost for a juvenile male (precocious male) may be much less but only a small number of eggs may be fertilised by that male.

The decision to mature precociously or remain immature for another winter has been modelled as a threshold trait [6,7]. A threshold level which takes into account parameters such as growth rate, body size and condition must be reached before the parr can mature. Both genetic [8] and environmental factors [9], which include social structure, are believed to play a part in attaining this threshold. For example, where environmental factors have been largely excluded by removing wild fish to a hatchery, the proportion of mature parr offspring was related to the source river of the parents suggesting involvement of a genetic component [10]. However, an environmental component was intimated by a study that showed tributaries of a single river had size thresholds for precocity that depended on the altitude of the tributary [11]. Precocity levels of 20% have been reported in Scotland [12] though levels as high as 65% have been reported for more southerly rivers [13] possibly as a result of the warmer waters and faster growth of the fish.

In cultured salmonids levels of precocity can vary depending on the genetic stock but high levels lead to reduced productivity and can present a health risk to the fish population through the negative effect of testosterone on immune function [14]. In the wild, fish that fortuitously find a better food source may grow faster and become socially dominant. Hatchery-reared fish are well fed yet growth and social hierarchy are still factors that influence the choice to mature precociously or undergo the parr-smolt transformation. In Ireland, wild Atlantic salmon generally remain in fresh water until the parr-smolt transformation at year 2+ or later [15]. They feed heavily in the marine environment for one year (grilse) or several years (multi-sea winter; MSW) before returning to their native streams to spawn. In the hatchery environment the majority of salmon parr are expected to smolt at year 1+: populations generally show a bimodal growth distribution in the previous autumn (year 0+) with larger fish preparing to smolt while the minor group of smaller fish will smolt a year later [16].

The juvenile male parr has three possible life trajectories: it can remain immature, it can undergo the parr-smolt transformation, or it can mature precociously prior to the parr-smolt transformation. Precocious parr are more likely to be those juvenile parr that have grown rapidly; they then transfer their energies into gonad development at the expense of bodily growth. At some point the balance will change when the immature parr out-grow the sexually mature parr and the social structure may again change [5].

The brain-pituitary-gonad (BPG) axis is the key regulator of sexual maturation. Neuron stimulation of the brain leads to stimulation of the pituitary through gonadotropin releasing hormone (GnRH) which releases relevant hormones, such as follicle stimulating hormone (FSH) and luteinising hormone (LH) into the blood plasma for transport to the effector tissue. In the testes LH induces the production of testosterone which then affects various aspects of male physiology, secondary sexual characteristics and behaviour. In this study our goal was to develop a comprehensive picture of gene expression in the gonad and brain in precocious males, and compare mature parr testes to adult testes. However, in an extension to this study, we also aimed to develop resources and investigate interactions between sexual maturation and the parr-smolt transformation. There are now several genomic resources available to the Atlantic salmon research community. The Canadian GRASP project [17], the Norwegian Salmon Genome Project http://www.salmongenome.no and the EU SALGENE project http://www.salmongenome.no/cgi-bin/sgp.cgi all have provided large numbers of cDNA sequences for the DNA databases. We have developed a new Atlantic salmon cDNA microarray that is specifically targeted at sexual maturation and the parr-smolt transformation: this is the first description of this array. The focus was on generating single tissue libraries of key transition stages from subtracted salmon cDNA libraries: we have observed that novel regulated genes are more likely to be represented in these subtracted libraries between two conditions than in single condition cDNA libraries [18].

Adult maturation differs from precocious maturation in a number of important aspects; adult fish mature in fresh water after a sustained period of growth at sea having undergone the parr-smolt transformation either one or several years earlier. We have therefore also considered whether some of the identified genes differed in expression between precocious males and returning males.

## Results and Discussion

### Microarray analysis of precocious testes

**Figure 1:**
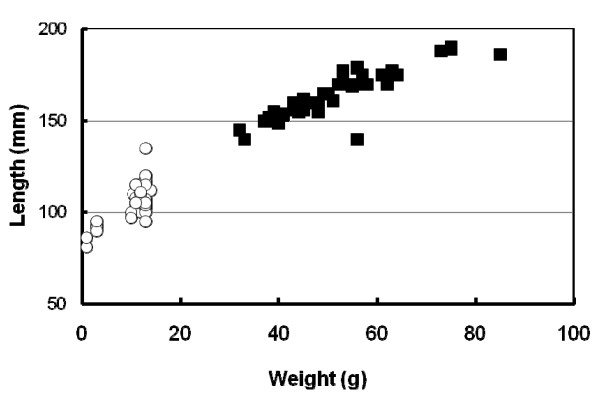
**Fish sample populations for precocity and non-precocity**. Precocious fish (empty circles) and non-precocious fish (full squares) were weighed and measured. Fish (25 of both precocious and non-precocious) were sampled on 15 Jan 2003. The difference in weight (using an unpaired t-test) and length (using a Mann Whitney test) in the two populations were both shown to be extremely significant (P < 0.0001).

Non-precocious parr were significantly longer and heavier than precocious parr (Figure 1). The difference in weight (using an unpaired t-test) and length (using a Mann Whitney test) in the two populations were both shown to be significant (P < 0.0001). It is generally accepted that males which grow at a faster rate are more likely to become precocious than slower growing male parr [19]. However, once this life history choice has been made (certainly by January), sexual maturation proceeds at the expense of somatic growth.

Total RNA, isolated from precocious and non-precocious parr testes was shown to be intact by both Northern blot and analysis using the Agilent 2100 Bioanalyser. All samples used subsequently had RIN values between 9 and 10 [20]. Suppression Subtractive Hybridisation (SSH) was used to enrich for genes differentially expressed during the process of precocious maturation [21]. Two pairs of libraries related directly to this study of precocity (testes and brain) while other SSH libraries related to different tissues (e.g. hypothalamus and pituitary) or different aspects of sexual maturation (e.g. adult maturing ovary). Furthermore, there was a selection of testes full-length (not SSH) and undifferentiated gonad (SSH) clones from other sources both salmon [22] and trout (McMeel and Guiguen, unpublished).

**Figure 2:**
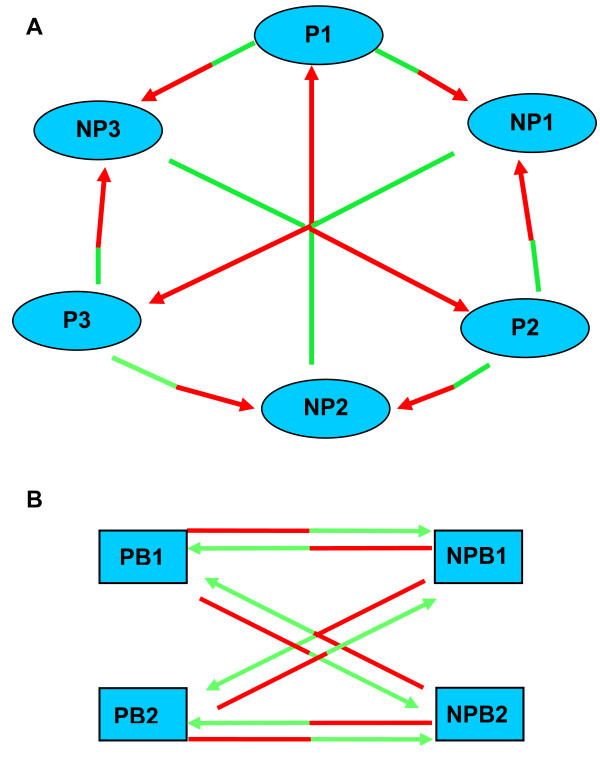
**Hybridisation designs for testes and brain microarray**. (a) Testes sample pools were hybridised using a loop design. Three precocious pools (P1-P3) each of four individuals were hybridised to three non-precocious pools (NP1-NP3) each of ten individuals. Each sample pool at the tail of the arrow (green) was Cy3-labelled and that at the arrowhead (red) was Cy5-labelled. Each pool was hybridised three times and at least once with each of the Cy5 or Cy3 dyes to maintain dye balance. Analysis was with LIMMA software. (b) Brain samples were hybridised directly using pools of five individuals. Pool labelling was as detailed above. Two separate pools of precocious brains (PB1 and PB2) were each compared directly to two separate pools of non-precocious brains (NPB1 and NPB2). All hybridisations include dye swaps. Analysis was with LIMMA software.

Three pools each of four individual precocious testes were compared by microarray analysis to three pools of ten pooled non-precocious testes (Figure 2a). Although not allowing a direct comparison of individual fish this loop design provided scope to look at individual pools yet maintain a compact design with dye balance and a minimum number of hybridisations [23,24]. This data series has GEO accession number GSE16721. A list of significant differentially expressed clones (P < 0.05 and using the Benjamini and Hochberg multiple test correction) was compiled. This list of 500 clones was cut down to approximately 65 for sequencing based primarily on fold changes in expression (Additional file 1: table s1). A shortened version of this table is provided as Table 1. Most clones (75%) showed homology to known database sequences, however 17% were found to have homology to unidentified ESTs and 8% 'failed' sequencing. 'Failed' sequences encompassed short sequences (e.g. too short to identify by homology searches), poor sequences (e.g. poly Ts and mixed clones), and no sequence (template problem). Between pool variation was also examined in LIMMA. For the P pools 484 of the 500 top clones were identified in all three pools and 499 in at least two of the pools. For the NP pools 499 of the top 500 clones were identified in all three pools and all 500 in at least two of the pools. This indicated that no individual sample was excessively influencing the overall precocious versus non-precocious comparison.

**Table 1:** Differentially expressed genes of the testis during precocious maturation.

**^1^GenBank Accession No**.	^2^Up or Down	Fold	Gene name	E-value	^3^Accession No. of Hit (Unigene if appropriate)
GO944178	**↓**	2.0	Alpha globin 1	0.0	NM_001124550

GO944180	**↓**	1.9	Alpha-4 globin	4E-178	BT050340

GO256596	**↑**	5.4	Anti-leukoproteinase precursor/OVP-2	6E-112	TC64926

GO256601	**↓**	7.2	Anti-mullerian hormone	0	NM_001123585

GO256604	**↑**	16.4	Apolipoprotein C-1	1E-153	NM_001141362

CR943959	**↑**	5.7	Apolipoprotein C-1	7E-129	NM_001141362

CR944033	**↑**	14.9	Apolipoprotein C-1	7E-129	NM_001141362

GO256602	**↑**	14.0	Apolipoprotein C-1	2E-141	NM_001141362

n/a	**↑**	6.6	Apolipoprotein E	n/a	CA050208

GO256613	**↑**	2.9	Apolipoprotein E	0.0	CB502338 (Ssa30932)

GO256585	**↓**	2.6	Beta globin	0.0	AY026061

GO944177	**↓**	3.2	Beta globin	0.0	YO8923

FD425634	**↓**	1.7	Beta-2 microglobulin	1E-64	TC33956

GO256603	**↑**	3.6	Cathepsin B	6E-134	TC55653

GO256605	**↑**	2.7	Cathepsin B	6E-134	TC55653

FD425625	**↓**	3.7	Collagen 1A2	6E-33	TC37297

FD425605	**↓**	3.7	Collagen 1A2	9E-100	TC22424

FD425653	**↓**	4.5	Collagen 1A2	9E-100	TC22424

GO256584	**↓**	1.8	Elongation factor EF-1 alpha	0.0	AF498320

n/a	**↓**	1.8	Elongation factor EF-1 alpha	n/a	CB502673 (Ssa.30552)

BI468075	**↓**	2.4	Elongation factor EF-1 gamma	1E-121	TC41574

n/a	**↓**	1.5	Elongation factor EF-2	n/a	DV196880 (Omy.33489)

FD425597	**↑**	2.9	GDP-mannose 4, 6-dehydratase	2E-73	TC26943

FD425627	**↑**	1.1	Glutamine synthetase	3E-44	TC28369

FD425678	**↓**	1.23	Glutathione S-transferase	6E-98	TC117527

GO944125	**↓**	2.1	Glutathione S-transferase	8E-116	TC111174

GT222009	**↓**	1.6	Growth hormone 1 precursor	0.0	CX719563

GO256590	**↓**	2.6	Guanine nucleotide binding protein/RACK1	0.0	BT043532

GT222008	**↑**	1.6	Heat shock protein hsp70a	0.0	NM_001124232

n/a	**↓**	1.7	Heat shock protein hsp90 beta	n/a	AJ632154 (Ssa.1060)

BI468080	**↓**	1.4	Heat shock protein hsp90 beta	n/a	BI468080.

FD425670	**↓**	1.6	Heat shock protein hsp90 beta	4E-24	TC22349

FD425588	**↓**	2.3	Heat shock protein hsp90 beta	0	AF135117

n/a	**↑**	1.9	Gonadotropin-releasing hormone receptor	n/a	CA046044 (Omy.8048)

GO256593	**↑**	2.7	Lipoprotein lipase	8E-77	TC52451

GO256597	**↑**	2.1	Nuclear Protein-1	0	BT048359

GO256611	**↑**	2.6	Proopiomelanocortine B	8E-81	DQ508935

GT145199	**↑**	2.3	Retinoic acid receptor responder protein 3	0.0	EG875900 (Ssa.836)

n/a	**↑**	2.0	16S Ribosomal rRNA	0.0	DQ864465

GT145267	**↓**	2.4	Ribosomal protein L8	7E-59	AY957563

FD425672	**↓**	2.4	Ribosomal protein S5	4E-89	AF543539

GO944179	**↓**	1.7	Similar to CD209 antigen-like protein D	0.012	BT048497

GO256582	**↓**	7.9	Similar to collagen 1A3	2E-36	TC151122 (O.mykiss)

GO256609	**↑**	1.4	Similar to influenza virus NS1A binding protein b	7E-12	BC066513

GO256612	**↑**	1.3	Similar to neural cell adhesion L1-like	8E-151	TC82871

FD425595	**↓**	2.0	Similar to ribosomal protein L5	9E-134	BT046397

FE963911	**↑**	1.4	Similar to SIX homeobox 6	1E-90	DW628904

GO256594	**↓**	6.0	Transferrin	3E-152	BT045182

FE963864	**↓**	5.2	Zinc finger protein Zic1	5E-143	NM_001140488

^1^Clones on this list may already have been sequenced in other projects (e.g. STRESSGENES). Accession numbers are given where available.

^2^An upward arrow indicates up-regulation in precocious males

^3^BLAST accession numbers starting with TC indicate sequences in the TIGR database.

From Table 1 it would appear that the protein translational machinery has been substantially down-regulated in precocious males: genes for the heat shock proteins (Hsp70a and Hsp90b), the elongation factors (EF-1alpha, EF-1gamma and EF-2) and the ribosomal proteins (RpL5, RpL8 and RpS5) attest to this. Cathepsin B was up-regulated in the maturing testes as too was an antileukoproteinase similar to trout ovulatory protein (OVP-2): this suggests regulated re-structuring of the gonad in a similar manner to what happens in the ovary. Apolipoprotein (Apo) E is involved in lipoprotein uptake and processing; it is a carrier for lipid into the cells and subsequently mediates the exchange of lipids and cholesterol in LDL. Lipoprotein lipase (Lpl), which digests plasma lipoproteins releasing lipids which apolipoproteins including ApoE can internalize [25], was also identified as an up-regulated gene in the precocious testes. Possibly ApoE is required for transport of cholesterol into the Leydig cell mitochondria in order to make available cholesterol for steroidogenesis or for transport of lipid needed either as a energy source or for cell membrane biosynthesis in gametogenesis [26]. Anti-Mullerian hormone (AMH: also known as Mullerian Inhibiting Substance) was strongly down-regulated in the precocious male testes. AMH has growth factor activity with a TGFbeta-like C-terminal domain and is known to have roles in Mullerian duct regression, sex determination, sex differentiation and gonad mesoderm development [27, 28]. Its role in testicular differentiation in fish was suggested when a spermatogenesis-preventing substance was isolated from eel [29] and further reports confirm significant regulation in other fish [30,31]. Another up-regulated gene was gonadotropin-releasing hormone receptor (GnRH-R). The expression of gonadotropin-releasing hormones (GnRH) and their receptors in the gonads of fish and many other species has been reported previously [32] and suggests paracrine/autocrine control on gonad development. Although in fish there are multiple forms of GnRH peptides and GnRH receptors there appears to be one form of GnRH and GnRH-R in the gonad which varies in expression considerably depending on reproduction status [33]. Increased GnRH-R1, as reported in this study, is associated with the later stages of spermatogenesis in rainbow trout [33]. Another gene more often associated with the pituitary that shows differential expression in the testes is growth hormone (GH). Down-regulation, as seen here, probably relates to the full maturity of these testes (January) and the higher reported levels of receptors for growth hormone in recrudescent testes [30,34]; if gene transcription is being switched off lower levels might be expected that in the immature parr where levels may be quite steady or rising in preparation for the parr-smolt transformation [35].

### Quantitative PCR on pooled precocious testis

**Figure 3:**
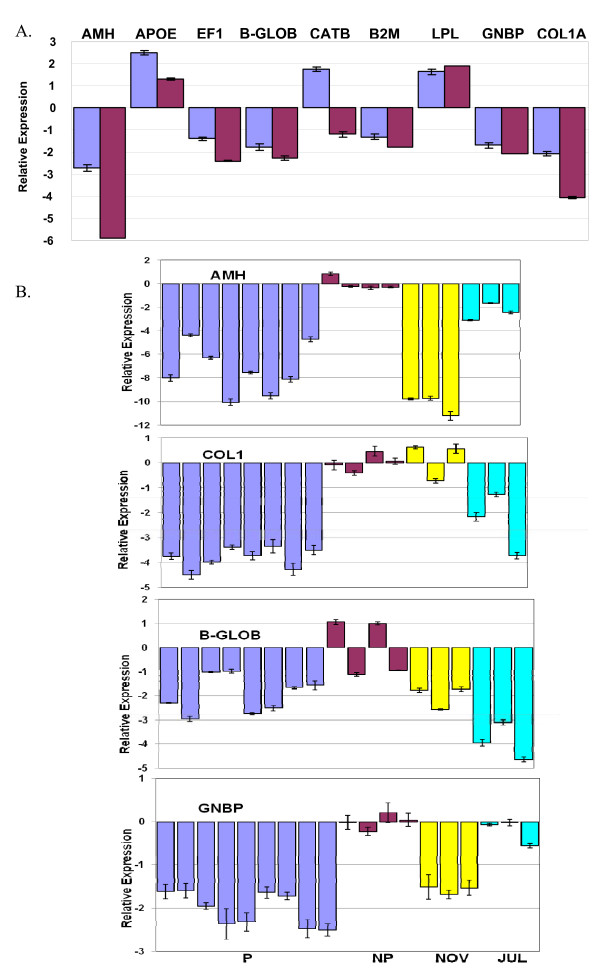
**Microarray and qRT-PCR data for select genes in testes samples**. (a) A comparison of microarray and qRT-PCR data in pooled samples. Microarray data (blue bars) for the LIMMA comparison of precocious and non-precocious testes were compared to qPCR data (red bars) for the same RNA pools that were used in the microarray analysis, i.e. pooled precocious (n = 12) and non-precocious (n = 30). Positive values are up-regulated in precocity and log2 values are plotted on a linear scale for clarity. All qPCR data was normalised using succinate dehydrogenase (SD) as reference gene. qPCR values are Means ± SEM of triplicate measurements. Error bars are only shown on the microarray data where the gene is represented more than once in the list of significant genes. (b) qPCR of individual testes samples relating to precocity and adult maturation. Samples of precocious testes (blue bars; n = 8-9), and July (turquoise bars) and November (yellow bars) maturing adults (n = 3) were of individuals whereas those of non-precocious males (red bars) were 4 pools each of 10 testes. Relative expression levels are plotted as log2 values on a linear scale. Succinate dehydrogenase (SD) was the reference gene in all cases. All primers were designed to the database sequence of the identified gene (see Table 4). Values are Means ± SEM of triplicate measurements. See Additional file 2 for some further details. AMH: Anti-Mullerian Hormone, APOE: Apolipoprotein E, EF1: Elongation Factor 1, B-GLOB: beta-globin, CATB: cathepsin B, B2M: Beta-2 microglobulin, LPL: Lipoprotein lipase, GNB2L1: Guanine nucleotide binding protein (G protein) beta polypeptide 2-like 1, COL1A: Type 1A collagen.

In order to confirm the findings of the microarray analysis on the testes tissue a number of genes were selected and further investigated using real-time quantitative PCR. These qPCR experiments were carried out on the same pools used for microarray hybridisations. Several potential housekeeping genes were analysed for use as a reference gene in quantitative PCR. Our goal was to identify a gene that could be reliably used as a reference in both the precocious samples and the adult maturing samples. Most of the genes examined (beta-actin, glyceraldehyde-3-phosphate dehydrogenase, hypoxanthine-guanine phosphoribosyl transferase, cyclophilin and ubiquitin) were significantly differentially regulated in at least one developmental stage, but changes in succinate dehydrogenase (SD) expression levels were not significant (P > 0.05) (Additional file 2). Relative expression was therefore calculated using the Delta Delta C(t) method [36] using succinate dehydrogenase (SD) as the reference gene. Preliminary quantitative PCR experiments were set up to analyse the expression patterns of the genes from the panel using biological pools of non-precocious parr testes and precocious parr testes (Figure 3a). Nine genes were selected for qPCR based mainly on the number of times the gene was independently identified on the array and on an examination of both fold changes and levels of significance. Of the genes selected only cathepsin B showed a discrepancy between microarray analysis and qPCR. It appears that the microarray SSH clone was similar to GenBank accession no. NM_001140522 (*S. salar *cathepsin B) but that the qPCR primers were designed to EST EG934102. This EST is also described as 'cathepsin B' but is a different isoform - different enough that the (reverse) primer is specific for this form. It is open to speculation why one form may be down-regulated more than 2-fold (qPCR) while, as the microarray suggests, another form appears up-regulated more than 2-fold. The fold changes in differential regulation were frequently larger by qPCR than by microarray: AMH levels by microarray analysis were ~6.5-fold down-regulated in precocity yet qPCR showed the fold down-regulation to be as much as 59-fold.

### Quantitative PCR on individual testis

After validation using fish pools, qRT-PCR of individual fish was carried out on a number of the genes (Figure 3b). As stated previously AMH was known to have roles in Mullerian duct regression and sex differentiation so it was an interesting candidate. Guanine nucleotide binding protein (G protein) beta polypeptide 2-like 1 (GNB2L1), also known as RACK1, plays a part in cellular signalling pathways [37] and is known to compete with Hsp90 for binding to hypoxia-inducible factor 1alpha (HIFalpha) [38]. Both alpha- and beta-globin were down-regulated in the testes in precocity and, like type 1 collagen (COL1A), they were identified more than once independently on the array. In order to add value to this data maturing adult testes sampled in July and November were also analysed: whereas November testes are likely to be almost mature (GSI ~ 2.4), July testes will be at a much earlier stage of maturation (GSI ~ 0.2). All expression levels were normalised to the succinate dehydrogenase reference gene (Table 2).

**Table 2:** Quantitative PCR validation of microarray analysis

Gene	Microarray	qPCR	qPCR
	**Testes Expression (Fold Change)**		

	**P relative to NP**	**P relative to NP**	**November relative to Jul**

AMH	-7.2	-58.9	-231.6
ApoE	+4.8	+2.5	+1.2
EF1α	-1.8	-5.3	-3.7
β-Glob	-2.9	-4.9	+3.7
Cat B	+3.2	-2.3	1.0
β2M	-1.7	-3.4	-2.1
LPL	+2.7	+3.7	+2.9
GNBP	-2.6	-4.3	-2.6
Col 1A	-4.0	-16.7	+5.8

	**Brain Expression**		
	
ApoE	-1.3	-1.4	
β-Glob	-1.5	+1.3	
IT-1	n/a	-9.0	
IT-2	-1.5	-9.1	
MCH	+1.2	+1.7	
02A07	-1.3	+1.1	
04H01^1^	+1.5	+1.2	
22D02	+1.3	+3.0	

^1^Clone included a microsatellite

AMH expression in individual precocious testes was down-regulated approximately 20- to 1070-fold in different individuals. Adult maturing testes followed a similar pattern where the more mature November adults showed high levels of down-regulation (850- to 2400-fold) and the less mature July adults showed only 3.1- to 8.6-fold down-regulation. There was more variability in the precocious levels of AMH expression than the November adults; though the number of the latter is very low (3), this might suggest that maturation is much more synchronised in the latter. AMH levels were also generally lower in the November adults than in the precocious fish - five of the seven precocious males had levels much higher than the three November adults.

Guanine nucleotide binding protein (G protein) beta polypeptide 2-like 1 (GNB2L1) showed a similar pattern of expression to AMH, with down-regulation in precocious parr and in November adults compared to non-precocious parr and July adults though fold changes in expression level were small, approximately 1/10 those of AMH. Collagen (1A) and beta-globin also showed down-regulation in precocious parr but July adult levels were lower than November adults. Statistical analysis by Kruskal-Wallis and Dunn's Multiple Comparisons tests demonstrated that expression in the precocious testes was significantly different from that in the non-precocious testes for all genes (P < 0.001; beta-globin beta P < 0.01) as it was also for July v November maturing adults. For AMH and GNB2L1 expression in precocious testes was similar to that of adult November testes and expression in non-precocious testes was similar to that of adult July testes (P > 0.05). For beta-globin non-precocious testes were significantly different from all other testes types (P < 0.01).

### Microarray analysis of precocious brains

Precocity effects on brain gene expression were also measured by microarray analysis. Here two pools of five precocious brains were compared directly to two pools of five non-precocious parr brains (Figure 2b). Data was analysed with LIMMA to determine which genes were differentially regulated between the pools for the two tissue types (PB v NPB). This data series has GEO accession number GSE16720. An initial list of 487 clones (P < 0.05) was reduced to a top 100 clones (on P-value) and 66 that were sequenced are presented (Additional file 3: table s2). A shortened version of this table is provided as Table 3. The fold changes in expression level were small compared to changes in testes expression with a maximum of 1.7 fold up-regulation for an unknown EST and 1.9 fold down-regulation for vasotocin-1. Approximately 50% of the top 66 clones were derived from brain-related libraries. Alpha-globin was identified five times on the array and beta-globin three times: both genes showed consistent down-regulation of 1.4 fold and 1.5 fold respectively. Other genes that were identified more than once included beta-tubulin (2×), elongation factor 1 alpha (3×), apolipoprotein E (2×), suppressor of G2 allele of SKP1 (2×) and vasotocin-1 (2×); the only inconsistency was that one copy of G2 allele of SKP1 was up-regulated 1.4× and the other copy was down-regulated 1.3×. Several of the clones represented definite ESTs which were highly homologous to other ESTs in the NCBI and TIGR databases but for which identities were ambiguous. Again between pool variation was examined in LIMMA. Of the 142 genes showing significant differential regulation (P < 0.01) between the precocious and non-precocious brain samples, 23 also showed significant differential expression between one of the biological replicates, i.e. PB1 v PB2 (12 clones) or NPB1 v NPB2 (11 clones). This is possibly a symptom of the small expression changes and subsequent limitations of the analysis; however 84% of the genes were still unique to the precocious versus non-precocious comparison. From a physiology perspective it could also be argued that the brain has multiple roles to play at any one time and is therefore more affected by the environment than the testes. Individual fish may thus, depending on slight variations in conditions such as social status, availability of food, etc., show more variation in brain expression than in gonad expression. Interestingly all eight globin clones were included in the 12 clone overlap with the PB1 v PB2 comparison and isotocin and both vasotocin clones were included in the 11 clone overlap with the NPB1 v NPB2 comparison.

**Table 3:** Differentially expressed genes of the brain during precocious maturation.

**^1^GenBank Accession No**.	^2^Up or Down	Fold	Gene name	E-value	^3^Accession No. of Hit (Unigene if appropriate)
BE518469	**↓**	1.5	Alpha-globin 1	0.0	NM_001124550

GO944156	**↓**	1.3	Alpha-globin 1	0.0	NM_001124550

BE518469	**↓**	1.3	Alpha-globin 1	0.0	NM_001124550

GR209362	**↓**	1.4	Alpha-globin 4	5E-101	BT050340

GO944180	**↓**	1.5	Alpha-globin 4	4E-178	BT050340

GT145313	**↓**	1.2	Anamorsin	0.0	BT043538

n/a	**↓**	1.3	Apolipoprotein E	n/a	CA050208 Ssa.30932

GO256613	**↓**	1.3	Apolipoprotein E	2E-123	TC91898

BE518484	**↓**	1.3	Beta-2 microglobulin	0.0	NM_001123699

n/a	**↓**	1.2	Beta-actin	n/a	NM_001123525

GO256610	**↑**	1.2	Beta-catenin interacting protein 1	0.0	NM_001139923

GO256585	**↓**	1.5	Beta globin	0.0	AY026061

GO944177	**↓**	1.4	Beta globin	0.0	YO8923

BE518474	**↓**	1.5	Beta-globin	0.0	NM_001123666

n/a	**↓**	1.2	Beta-tubulin	n/a	NM_001139793

GO256580	**↓**	1.3	Beta-tubulin	2E-103	NM_001140292

CR943970	**↓**	1.3	Beta tubilin	8E-54	BT058720

n/a	**↓**	1.3	Cathepsin D	n/a	NM_001124711

FE963874	**↓**	1.2	Cofilin 2	2E-94	BT060221

n/a	**↓**	1.2	Elongation factor EF-1 alpha	n/a	CB502673

GO256584	**↓**	1.2	Elongation factor EF-1 alpha	4E-152	BT058929

GO256588	**↓**	1.2	Elongation factor EF-1 alpha	0.0	NM_001141909

GO944131	**↓**	1.1	Ferritin H-1	3E-112	NM_001124547

n/a	**↓**	1.2	GAPDH	n/a	NM_001123561

n/a	**↓**	1.2	GAPDH	n/a	NM_001123561

n/a	**↓**	1.2	Glutamine synthetase	3E-10	NM_001124314

GO256590	**↓**	1.2	Guanine nucleotide binding protein, beta polypeptide 2-like 1	0.0	BT059305

GT222010	**↑**	1.2	Heterogeneous nuclear ribonucleoprotein A/B	2E-106	BT045277

n/a	**↑**	1.2	Hydroxysteroid 11-beta dehydrogenase	n/a	AB104415

BE518475	**↑**	1.2	Interleukin enhancer binding factor 2	1E-70	BT045275

n/a	**↓**	1.5	Isotocin 2	n/a	NM_001123652

n/a	**↑**	1.2	Melanin-concentrating hormone 2	n/a	M25755

GT145268	**↓**	1.2	MHC class I	0.0	AF504013

GT145243	**↓**	1.2	MHC class I	0.0	AF504016

FE963873	**↓**	1.2	^4^Myelin P0-like glycoprotein	7E-90	NM_001140077

GR209337	**↓**	1.2	Myosin-9 (putative)	0.0	BT072044

GO944099	**↑**	1.2	Phosphogluconate dehydrogenase	2E-94	BT059099

CR944012	**↓**	1.3	Phosphoglycerate mutase 1	2E-64	CX249369 (Omy.36005)

GO256580	**↑**	1.2	Proline-rich nuclear receptor coactivator 2	0.0	NM_001140247

CR944197	**↑**	1.3	Reverse transcriptase-like protein	0.0	CR944197

BM413842	**↓**	1.2	60S Ribosomal protein L13A	0.0	BT044039

CR943861	**↓**	1.2	S100 calcium binding protein	0.0	NM_001146376

BM413745	**↓**	1.2	Simple type II keratin K8	0.0	NM_001124734

BF228584	**↑**	1.4	Suppressor of G2 allele of SKP1	0.0	BT071868

BF228584	**↓**	1.3	Suppressor of G2 allele of SKP1	0.0	BT071868

n/a	**↑**	1.2	TGFB2	n/a	Aj318936

GO256586	**↓**	1.2	VAMP-2	2E-176	CA060527 Ssa.21646

n/a	**↓**	1.7	Vasotocin-1	n/a	DY736376

n/a	**↓**	1.9	Vasotocin-1	n/a	DY736376

See footnotes to Table 1. In addition:

^4^First 539bp do not align to myelin P0-like glycoprotein (S. salar): 318/321 (99%) align to S. salar EST GE77456

### Quantitative PCR on pooled brains

In order to validate the findings of the microarray analysis on the brain tissue a number of genes were selected and further investigated using real-time quantitative PCR. These qPCR experiments were carried out on pools of five brains (either PB1 or PB2 against NPB2). The reference gene used was ubiquitin (Additional file 2). Changes suggested by microarray analysis were generally confirmed by qRT-PCR, however, unidentified clone 02A07 and beta-globin, both of which appeared down-regulated on the array, were shown to be relatively unchanged by qPCR (Table 2). Melanin-concentrating hormone (MCH), which was in the top 100 list, appeared to be up-regulated on the array in precocity. This gene was examined in 5 precocious individuals (PB2 pool) and in 5 non-precocious individuals (NPB2 pool). On average MCH was up-regulated approximately 1.7 fold in precocious males.

Although many studies of male precocity in salmonids have reported the association of physical features of fish with life trajectories [4,5] it is only recently that a number of papers have reported the use of genomic approaches to investigate genetic changes associated with these life trajectory decisions in salmonids [39-42] and other species [43,44]. The array described herein has been generated from clones which have been selected in relevant tissues for their role in processes that are likely to be involved in precocity. SSH is a commonly used molecular technique to enrich for genes differentially expressed between two populations [21]. Here it has been used to enrich for precocity- and maturation-related genes in brain, brain-related and testes tissue. Furthermore, as the parr-smolt transformation and sexual maturation are often considered competing processes, smolting SSH libraries (for brain and related tissues) may also be relevant to precocity.

In all 500 genes were determined to be differentially expressed between precocious and non-precocious gonad pools (P < 0.05) and 142 genes between precocious and non-precocious brain pools (P < 0.01). In both cases there were approximately 3 times more genes down-regulated than were up-regulated. This suggests a turning down of non-essential processes and the turning up of specific pathways. Some patterns of expression suggested the involvement of specific physiological processes. However, both the relatively small number of unambiguously identified genes and the paucity of GO terms for these meant that a thorough GO analysis was not possible. Gene expression changes in the brain were small but this is reflected by other papers on brain expression [45-47]. It is a common observation that brain expression changes are often less than 2-fold which has been noted in fish [45,48] and in other species [49,50]. Microarrays have been reported before to show smaller changes in expression than the more robust technique of qPCR and indeed we have found this ourselves in related studies [51]. Alternatively, the microarray signal represents all targets that hybridise to the probe whereas qPCR being primer-based may distinguish between different targets and be more discriminatory.

Apolipoprotein E was down-regulated in the brain whereas it had been up-regulated in the gonad (both confirmed by qPCR). It is unlikely that the specific role of ApoE is the same in both brain and testes. ApoE is involved in neural regeneration of the peripheral nervous system through the redistribution of cholesterol [48] therefore it is possible that it is in this role that ApoE affects brain development in the precocious male. Down-regulation of both isotocin (confirmed by qPCR) and vasotocin was evident in the brain of precocious male fish, however all three clones representing these genes on the array were also differentially expressed between the two NPB pools (P < 0.01). This suggests some important variation in the ten non-precocious individuals that made up the two pools. Up-regulation of vasotocin, and to a lesser extent isotocin, is more often associated with reproduction especially through the known effects of vasotocin on social behaviour [52,53]. However, differences in the effects of vasotocin administration on aggression and/or courtship are clearly seen between fish species with some showing increased and others decreased aggression [54-56]. There are also seasonal variations in transcript levels of both vasotocin and isotocin in, for example, the masu salmon which in addition show changes between immature parr and precocious males [57]. It has been observed that the protein levels of these hormones do not correspond to the levels of their mRNAs - vasotocin transcript levels are low in Nov in immature parr when vasotocin protein levels are near maximal [57]. Furthermore, vasotocin is also involved in osmoregulation, cardiovascular activity, stress and metabolism [58]. Because precocity possibly interferes with normal progress through the parr-smolt transformation, the relative decrease in vasotocin we observe in precocious fish may be linked to an early regulatory effect on osmoregulation, and the variation we observe in the non-precocious individuals may be due to early preparation for smolting in some fish.

Another gene that appeared to be up-regulated (1.2-fold) in the brain was melanin-concentrating hormone (MCH). MCH is expressed as a pro-hormone mainly in the hypothalamus and processed in the pituitary where the mature short peptide (17 amino acids in most teleosts) stimulates the aggregation of melanosome in melanophores resulting in the silvering of the fish [59]. This is somewhat at odds with the expected inhibition of silvering in precocious fish where the parr-smolt transformation is delayed. Receptors for MCH are, however, distributed much more widely than the brain and hypothalamus suggesting that the functional roles of MCH (which may include regulating food intake) are much more diverse than melanin concentration [59,60]. The DNA sequences of the MCH1 and MCH2 isoforms of Chinook salmon MCH are very similar and are unlikely to be distinguished on the microarray. Because both MCH isoform sequences are not available for Salmo salar, two fragments were isolated and sequenced from Salmo salar cDNA using primers based on the Chinook sequences [61]. Quantitative PCR (using primers designed to the two Atlantic salmon fragment sequences) of individual samples from the PB2 and NPB2 pools showed approximate 1.7-fold up-regulation of MCH1 and 1.4-fold up-regulation of MCH2 in the precocious samples. MCH2 levels (C_t_~34) were at least 100 times lower than MCH1 levels (C_t_~26) so comparisons of MCH2 levels between individuals were not reliable; however there was still considerable variability in the levels of MCH1 in the ten individuals. An approximate 290-fold difference (Additional file 2) between the highest and the lowest individuals may reflect sampling inconsistency where some specialised regions of the brain may have been mistakenly included (although the pituitary and hypothalamus had been purposefully dissected from all brain samples).

Alpha- and beta-globin gene expression was down-regulated in the precocious brain as they had been in the testes. Surprisingly all eight globin clones were also differentially expressed between the two pools of precocious brains (PB1 and PB2); this was eight of the nine sequenced clones in a list of twelve clones (P < 0.01). Although the melting point curve on qPCR showed a single peak, it was noticeable during the optimisation of beta-globin primers that there were at least two forms amplified. Haemoglobin multiplicity in teleosts is common: embryonic forms give way to adult forms and it also has been suggested that fish which travel great distances across temperature differentials show more multiplicity [62].

There are some interesting similarities and differences between our precocious males and those of Aubin-Horth [39,41]. Both studies show down-regulation of genes in precocious males was more pronounced than up-regulation; we see down-regulation of glutamine synthetase and RpL13A which ties in with the above study but we also see down-regulation of vasotocin, isotocin and type II keratin K8 which seems at odds with the Aubin-Horth study. Also alpha- and beta-globin are oppositely regulated in both studies. Possibly the differences seen in the two studies relates to the different times of fish sampling which were October for the Aubin-Horth study and three months later in January for our study. We chose to collect both groups of males in January when precocity was externally evident (size, body shape, milt production). In January the testes could therefore be best described as mature, possibly on the point of regression whereas in October they are likely to be recrudescent. Since maturation is controlled by the HPG axis, in October the brain will be signalling the gonads to mature and to begin steroidogenesis, and accompanying this there will ensue a range of behavioural changes. Therefore it is possibly not surprising that the Aubin-Horth study shows up-regulation of genes involved in reproduction and this study shows some opposite effects.

Furthermore, our control group (immature males) may be preparing for the parr-smolt transformation (transfer in the first week of May). There may therefore be some changes in brain precocity expression which are confounded by brain smoltification expression. In a separate experiment (White et al., personal communication), the same genes were followed in a brain smolt time-course from January to May. From this it is clear that most of these genes do not change significantly over the period of smoltification and therefore it is unlikely that down-regulation in precocity is really up-regulation in smoltification. It is however interesting that in the Aubin-Horth study several genes, including beta-actin, cathepsin D, an elongation factor, beta tubulin and both vasotocin and isotocin, showed relative up-regulation in 'early migratory' fish that also showed up-regulation in our immature parr.

The fish used in this study are from a hatchery-reared stock that has been developed from a small number of originator males and females and should therefore be of relatively homogeneous genotype. We do not necessarily expect that hatchery and wild salmon will behave in the same way. It has been shown that the rearing environment of Atlantic salmon has an effect on gene expression and additionally that this effect interacts with mating tactic [39]. In a study that used a cichlid microarray to compare wild and laboratory reared salmon from the same source it was clear that some differences in expression between immature male brains and precocious brains were dependent on the rearing environment [39]. This in itself suggests that the sneaker tactic/phenotype does not develop by one unique route and therefore that gene expression profiles may differ from one individual to another due to environment. Also it must always be remembered that for many regulators such as isotocin and vasotocin, because of storage, secretion and blood clearance considerations, transcript levels in the brain and gonad may not directly correlate with protein levels in the plasma, and that plasma levels do not necessarily reflect activation of the system as the hormone receptor in the effector tissue may be down-regulated to prevent effects [57]. As noted above, isotocin and vasotocin blood plasma levels may be high leading to activation of a pathway or process but transcript levels could be low because either the hormone was released from stores (not requiring *de novo *transcription) or a feedback mechanism was down-regulating transcription. It is a complicated issue where the specific biology is known to vary between different fish species and even within species of the family Salmonidae

## Conclusions

A custom-built salmonid cDNA microarray has been used to compare expression changes in the brain and gonad during the process of precocious sexual maturation in Atlantic salmon. Transcriptional regulation in the brain was minor compared to the testes. Microarray analysis suggested a general down-regulation of housekeeping functions and more specific up-regulation of a number of genes. Distinct expression profiles for both brain and gonad will be determined by maturation stage (recrudescent, mature, regressed) and environmental factors. Anti-Mullerian hormone plays a major role in the development of the maturing gonad as too does lipid mobilisation through the apolipoproteins and lipoprotein lipase.

## Methods

### Sample collection

The Marine Institute in Newport, Co.Mayo, provided Atlantic salmon samples. The Marine Institute, Furnace, Newport, Co. Mayo is a registered premises under the Cruelty to Animals Act 1876, as amended by EU Regulations 2002 & 2005 and is declared to be a registered place for the performance of experiments. The Burrishoole ranch stock used in this study were originally derived from Burrishoole wild stock and have been line bred since the early 1970's as part of an on-going experimental ranching programme.

At sampling, fish were transferred to an anaesthetic/sedative solution (ethyl 4-aminobenzoate; 0.1 g/l; Sigma-Aldrich) and killed by spinal section. Fifty precocious male parr and fifty non-precocious male parr (sex determined on dissection) were sampled in January 2003 to provide brain and gonad tissue for SSH library construction. Length and weight measurements were taken. Additional fish for microarray and qRT-PCR analysis were harvested in January 2004. Adult salmon were sampled from upstream traps located on the Burrishoole river system on 31 July 2003 and 25 November 2003 - these were tagged therefore are hatchery-reared fish (not wild). The gonadosomatic index (GSI) - the ratio of the weight of the gonad to that of the whole fish (× 100) - for the July fish was 0.19 ± 0.04 standard deviations (SD) and for the November fish was 2.44 ± 0.45 SD. All tissues were removed to RNAlater or snap frozen in liquid nitrogen. Samples were stored at -80°C until RNA extraction.

### Extraction and analysis of RNA

All samples were lysed and homogenized in TRIzol reagent (Invitrogen Life Technologies, Paisley, UK) [63]. Qiagen RNeasy midi columns and wash buffers (Qiagen Ltd., West Sussex, UK) were used to purify the RNA. All samples were DNase treated on-column using the Qiagen DNA-free DNase Kit (Qiagen Ltd., West Sussex, UK). The concentrations of the RNA samples were determined by UV spectrophotometry (Shimadzu UV-1601 or Eppendorf BioPhotometer) and checked for quality using the Agilent Bioanalyser-derived RNA integrity number (RIN) (Agilent Technologies Ireland Ltd., Dublin, Ireland) and by denaturing formaldehyde gel electrophoresis followed by northern blotting and subsequent analysis with a radiolabelled beta-actin probe.

### Construction of Suppression Subtractive Hybridisation (SSH) cDNA libraries

Tissues used for the SSH libraries were brain, pituitary, hypothalamus, head kidney, spleen, intestine, gill, testes and ovary. Libraries related to this paper were from brain, pituitary, hypothalamus and testes tissues comparing male parr to precocious parr. Other SSH libraries of relevance compared the ovary of maturing adult females (July) and near maturity females (November). Other SSH libraries to be described elsewhere relating to the parr-smolt transformation were of brain (Feb v sea cage), hypothalamus (Feb v Apr), pituitary (Feb v Apr, Apr v sea cage), gill (Apr v May), and intestine, head kidney and spleen (Feb v May). Exact dates were 13 Feb, 4 Apr, 2 May and 27 May (sea cage) 2003. An additional library pair related to the post-blastula transition of the salmon embryo.

For testes tissue pools of precocious parr and non-precocious parr total RNA were each prepared from 10 individual fish. For brain tissue pools of precocious parr and non-precocious parr total RNA were each prepared from 4 individual fish. For hypothalamus tissue pools of precocious parr were prepared from 3 individual fish and non-precocious parr from 3 pools each of 3 fish (29 Jan). For pituitary tissue pools of precocious parr and non-precocious parr total RNA were each prepared from 10 individual fish (29 Jan and 13 Feb respectively).

The ovarian library from maturing adults and the brain library from smolting parr were made directly from mRNA isolated from total RNA using the Oligotex mRNA Isolation Kit (Qiagen, UK). All other libraries were made from SMART cDNA generated by reverse transcribing 1 μg of total RNA as detailed in the SMART cDNA Synthesis Kit (BD Biosciences, Oxford, UK). The PCR-Select cDNA Subtraction Kit (BD Biosciences, Oxford, UK) was subsequently used to prepare both forward and reverse subtracted libraries from each tissue. Subtracted target cDNA products were ligated into the pCR2.1 TA Cloning Vector (Invitrogen Life Technologies, Paisley, UK) using T4 DNA ligase (New England Biolabs) according to the manufacturer's instructions. Ligations were transformed into Ultracomp™ One-Shot Top 10F' chemically competent *E. coli *cells (Invitrogen Life Technologies, Paisley, UK). Recombinant white clones from each library (four 96-well plates for each library) were randomly selected from the plates, cultured in LB broth in 96-well plates and frozen in glycerol for archival storage at -80°C. Clones were PCR amplified using 2.5 units of Biotaq (Bioline, UK) in a total volume of 75 μl detergent-less buffer (30 mM Tricine, pH 8.0 containing 50 mM KCl, 2 mM MgCl_2_, 0.2 mM dNTPs and 0.2 μM of each SSH nested primer). PCR products were checked for single products on ethidium bromide stained agarose gels and spotted directly.

### Microarray design and construction

The cDNA microarray contained clones from a number of Suppression Subtractive Hybridisation (SSH) cDNA libraries [21] constructed from different salmon tissues at different developmental stages related to sexual maturation, smoltification and embryo development. Of the 5376 cDNA clones, 4128 were newly generated subtracted cDNA clones and the remaining 1248 clones were other SSH or full-length clones from related salmonid projects. All SSH clones average 600-900 bp in length. Several candidate genes and controls were also included. At the printing stage DNA sequences were only known for those clones that were used to check library quality/redundancy and for those clones that originated from related salmonid projects. Otherwise clones were only sequenced after microarray analysis had identified a differentially expressed gene. The top 100 clones approximately from each of the two microarray analyses detailed here, and the same for additional analyses to be detailed elsewhere, were sequenced (AGOWA, Berlin, Germany). Sequences were edited to remove vector and adaptor sequences, and analysed by BlastN and BlastX algorithms against the NCBI databases of non-redundant nucleic acids and the protein and EST accession databases http://www.ncbi.nlm.nih.gov/BLAST/[64]. BlastN was also carried out against the TIGR database http://compbio.dfci.harvard.edu/tgi/cgi-bin/tgi/Blast/index.cgi.

**Figure 4:**
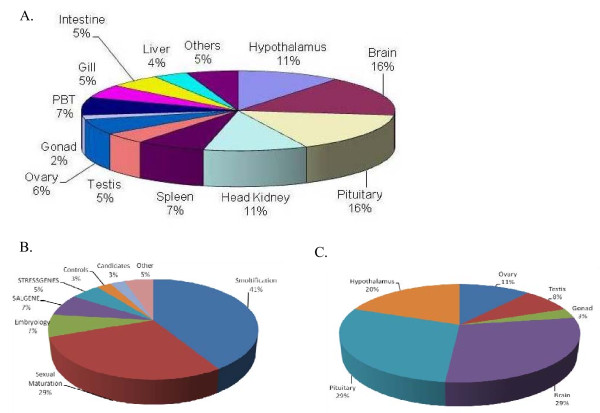
**Composition of the salmon maturation and smoltification cDNA microarray**. The array is described on the basis of the clone origin: (a) by tissue type, (b) by developmental stage and project source, and (c) by tissue library related to sexual maturation. Most clones originate from salmon SSH libraries related to sexual maturation and parr-smolt transformation, however 576 clones are of rainbow trout origin and relate to confinement stress in liver, brain and pituitary (European Union-funded STRESSGENES project, http://www.irisa.fr/stressgenes/), undifferentiated gonad and stimulated head kidney leucocytes (both included under 'Other'). A number of clones are also from unselected salmon full-length cDNA libraries of testis, ovary, spleen and liver (European Union-funded SALGENE project). Further details are given in Materials and Methods.

The printing onto amino-silane coated glass slides was carried out at the Liverpool Microarray Facility (University of Liverpool, UK) and consisted of duplicate arrays with sub-arrays in a 4 × 4 format with 336 spots per sub-array. The composition of the microarray by tissue and developmental stage is given in Figure 4. The array platform has been submitted to Gene Expression Omnibus with accession numbers GPL8731 (10752 features) and GPL8704 (5376 features): the duplicate features in the gonad analysis were not averaged where they were for the brain analysis, hence the two platforms.

### Hybridisation design

Non-precocious parr testes were small and collected in pools of ten. Precocious testes were more substantial and were RNA extracted individually. To compare testes samples precocious parr testes RNAs were pooled in three pools of four individuals and compared in an optimised loop design http://www.math.rug.nl/~ernst/book/smida.html[65] to three pools of ten non-precocious parr (Figure 2a). Nine microarray hybridisations were carried out in total as part of this design. To compare brain samples total RNA from 10 individual brains (dissected for hypothalamus and pituitary) of precocious fish and 10 brains of non-precocious fish were pooled to give two pools each of five precocious (PA and PB) and non-precocious (NPA and NPB) brains. Each pool of precocious brains was compared in a dye swap experiment with each pool of non-precocious brains (Figure 2b).

An indirect labelling method was used to attach Cy5 and Cy3 dyes to the cDNA. All target cDNAs were synthesised in one round. 10 μg of both tester and reference total RNA was used per hybridisation. cDNA was generated by reverse transcription using an anchored oligo VdT_26_, random primer (V9) and Stratascript RT enzyme. Amino-allyl (aa) dUTP/dNTPs produced from a 20× 1:1 stock of dUTP/dTTP were incorporated into the cDNA. This 1:1 ratio of dUTP/dTTP proved to be an effective ratio for the testes tissue. Contaminating enzymes and buffers were removed by purification with the Nucleospin PCR Clean-up Kit (Macherey-Nagel, Ger). Eluted cDNA was concentrated using a Vacufuge Concentrator 5301 at 42°C (Eppendorf, Ger). The resulting cDNA pellets were resuspended in 5 μl of sodium bicarbonate (0.1 M) (Sigma Aldrich). Cy dyes were resuspended in the same buffer and coupling to the Cy dye ester took place in the dark for 2 h at room temperature. The removal of unincorporated dye was performed using the Illustra CyScribe GFX Purification Kit (GE Healthcare). After coupling the amino-allyl (aa) dUTP-labelled cDNA to the Cy dyes (Amersham Pharmacia Biotech, UK), the labelled cDNA was again purified to remove any un-coupled Cy dye (Macherey Nagel, Ger). Slides were pre-hybridised in 50% formamide, 5× SSC, 0.1% SDS, 0.1 mg/ml BSA at 42°C for 1 h, then washed twice for 5 min with 0.1× SSC and for 30 s with 18 MΩ H_2_O. The slides were dried immediately by centrifugation (Eppendorf Multipurpose Centrifuge 5804) at 1500 rpm for 5 min at room temperature in a slide box lined with 3 MM Whatman paper. The denatured Cy dye-labelled cDNA was immediately applied to the slides. The purified, combined Cy5- and Cy3-labelled cDNAs were denatured at 95°C in a thermal cycler for 3 min. Dust was removed from the surface of the pre-hybridised arrays using an ozone-free air duster (Kenair, UK). Denatured target cDNA (80 μl) was applied to the array surface which was covered with a lifter slip (Erie Scientific, USA). Each array was placed in a pre-warmed Genetix hybridisation chamber. The chamber was sealed and incubated in an oven for 16-18 h at 50°C. Microarray wash solutions were warmed to 55°C. All wash steps were carried out in the dark. Each array was dipped in a 50 ml conical tube filled with wash solution 1 (2× SSC, 0.1% SDS) to dislodge the cover-slip. The arrays were washed twice for 5 min on a rotary table in fresh wash solution 1. The arrays were transferred to wash solution 2 (0.1× SSC) and washed twice for 1 min on a rotary table. Finally, the arrays were rinsed by immersion in RNase/DNase-free water to remove any residual salts, followed by brief immersion in 100% isopropanol (Sigma-Aldrich) at room temperature and brief centrifugation at 1500 rpm for 2 min to dry the slides. The arrays were scanned as soon as possible after washing.

### Microarray scanning and analysis

After hybridisation, fluorescence was measured using a confocal laser scanner (ScanArray Express HT, Perkin Elmer, USA). The high-resolution images were inspected using Genepix Pro v5.1.0.16 (gonad data) or Genepix Pro v6.0.1.25 (brain data) (Molecular Devices, USA). The LIMMA package for analysis of gene expression microarray data was used for both experiments [23,24]. For this spots were weighted based on feature flagging in Genepix: weak signal and feature irregularity appeared to be the main source of discrepancy therefore features were flagged to avoid these spots. Poor quality spots were assigned a weight of 0.1 whereas remaining good quality spots were given a weight of 1.0. For the gonad analysis median local background values were subtracted directly from median feature values, dye channels were swapped as appropriate and a 'printtiploess' normalisation (within slide) was applied. For the brain analysis background subtraction used the "normexp" method in LIMMA with an offset of 50, otherwise the treatment of data was the same as for the gonad experiment. LIMMA uses an empirical Bayes method for improved power in designs which only include a small number of arrays [23]. Different design models in LIMMA allowed a global comparison of the precocious and non-precocious gonads but, in addition, the different pools (i.e. 1 to 3) could be compared to each other: this was of interest as it could highlight any variation (either technical or biological) between individual samples. For the gonad analysis we chose to treat duplicates individually and only where both spots were significant (P < 0.05) was the spot accepted. This will tend to increase the number of false negatives but decrease the number of false positives. For the brain analysis we chose to average the MA values for duplicate spots.

### Quantitative PCR

Real-time PCR using SYBR Green-based detection was carried on the Mx3000P Real-Time PCR System (Stratagene). PCR primers (MWG Biotech, Ger) were designed according to the recommended guidelines in the QuantiTect SYBR Green PCR handbook (Qiagen Ltd., West Sussex, UK) and are provided in Table 4. All primer products and PCR conditions were first optimised using standard PCR with electrophoresis on ethidium bromide-stained agarose gels. cDNA was generated by reverse transcription of 1 μg total RNA (whether as a pool or individual) using an anchored oligo VdT_26 _random primer (V9) and Stratascript RT enzyme in a total volume of 20 μl following the manufacturer's protocol. cDNA was diluted to 100 μl before use as a template for qPCR. Initial analysis of gene expression was carried out on pooled individual cDNA samples. Technical variation was minimised by reverse transcribing all samples at the same time. The analysis was then carried out on individual cDNA samples in triplicate for each gene of interest. PCR data (as threshold cycle (Ct) numbers) were imported into Excel where the 2^-DeltaDeltaCt formula [36] was applied. This data was then imported into the GraphPad InStat programme (version 3.0) for statistical analysis. (Additional file 2).

**Table 4:** Primer list

Gene	Accession	Primer species	Primer	Primer sequences
Anti Mullerian Hormone	AY722411	S.salar	AMHF	ACAAGTGTTCGATCCAGACGTGAC

			AMHR	CACTCAGTCTGCCTTGGTGTGG

Elongation factor 1 alpha	AF321836	S.salar	EF1aF	TAAGGGCAACAGCAGTGGCAGTG

			EF1aR	CGCATTTGTAGATCAGATGGCCG

Beta-globin	BT050121	S.salar	BGF	CCCATGGCTGCGACAACCACTTTC

			BGR	CAACACACTCTTCGTCGACCCTGAC

Beta-2-microglobulin	NM_001123699	S.salar	RTB2F	GTACTTGTGCTCATTTACAGCGCGG

			RTB2R	GCCACTCACGTGACAGATCAGGG

^1^Type 1 collagen	BE518482	S.salar	T1C1F	GAGGCAATGACCGATGGCTTC

			T1C1R	GCGATGCTGTTCTTGCAGTGG

Cathepsin B	EG934102	S.salar	CatBF	TGTGAGACTGGATACACACCTGGCTAC

			CatBR	GCTCCTTCCACAGGTCCGTTCTTC

Guanine nucleotide binding protein	BT048931	S.salar	GNBPF	GTCGCCAAAATGACCGAGCAG

			^2^GNBPR	GTCTCATCACGGGTCAACTTCCAC

Lipoprotein lipase	EG868885	S.salar	LIPF	CGGCCCGACCTTTGAGTTTG

			LIPR	TTGGGGTAGATGTCCACGTGGC

Apolipoprotein E	NM_131098	D. rerio	ApoEF	TCTCTTGTGGTATTCTTTGCCCTGG

			ApoER	GTTCAGACACATACTGCCAGAAACGG

Calponin 3	BC053309	D. rerio	Cal3F	GCAACTGTGATTTTACTGCAACTTTAGGAC

			Cal3R	CAATCTTGTTTTTCACCTCCGCG

Isotocin 1	BT049938	S.salar	SsIT1F	AAAGCCTCAACCTCAACACATGGC

			SsIT1R	CTGGATGGGAAAGCTAGTGCTGA

Isotocin 2	CA063528	S.salar	SsIT2F	TCAGTAAATGGGTGGGTGAAATAGGTG

			SsIT2R	AGCTGCAAGTGACGCCAGGGT

02A07	FD425665	S.salar	02A07F	ACCATGTTGACAGCCAGTTTGCG

			02A07R	TTCCGCACTCTCAAGCTCACCAC

04H01	GT145175	S.salar	04H01F	ACTGTAGTAGTCAGCTTGTGAAGGATGC

			04H01R	GTAGTTCCATAGACAGAGAATGGATGCTC

22D02	FD425557	S.salar	22D02F	CAGCTGCTAATACAGGGTTATTGTTTTG

			22D02R	CACGTTTATGACAACTGACACGTG

^3^MCH1	M25754	O.tshawytscha	MCH1F	AAGAGGCCGACCAGGACCTGA

			MCH1R	ACTCAAGATGAGGCAGGACAAGATGC

MCH2	M25755	O.tshawytscha	MCH2F	TCCCCATCGGAAAGACGGAG

			MCH2R	TTCAGGTTCATCCACAGGCCA

Ubiquitin	BT060103	S.salar	UBQF	CCACAAAAAGCACCAAGCCAAC

			UBQR	AGCTGGCCCAGAAGTACAACTGTG

Beta-actin	NM_001123525	S.salar	ACTF	ATGGAAGATGAAATCGCCG

			ACTR	CCCTCTTGCTCTGAGCCTCG

Succinate de-hydrogenase	NM_001141694	S.salar	SUCDEF	GAGGGAAAGGAACAATACCATCAGACTG

			SUCDER	GACAGCAGGTCCCAGGTACTTGTCTC

^1^BE518482 is collagen 1A1

^2^GNBP: there is one mismatch here to the salmon sequence

^3^MCH1 and MCH2 nomenclature is based on the Chinook salmon (O.tshawytscha) MCH sequences

## Authors' contributions

AG and DF carried out the microarray studies. AG, DF, SH, AMOK, SLW and PFF were involved in the weighing, measurements and gonadal index determination of the precocious fish. AG, DF, SH, AMOK, SLW and PFF prepared and characterised the SSH libraries. AG, BH and PD carried out the qRT-PCR. DC provided the fish, the facilities to dissect these and background expertise on these fish populations. SH and AMOK organised the logistics of the array construction. OLP provided software and bioinformatics help throughout. TJS was academic supervisor to AG and guided her role in the work. MTC and NPW conceived of the study, and participated in its design and coordination and drafted the manuscript. MTC carried out all LIMMA analysis. All authors read and approved the final manuscript.

## Supplementary Material

Additional file 1 **Full version of Table 1 (Table 1s)**. Differentially expressed genes of the testis during precocious maturation. This version includes additional clones that were not definitively identified and includes two additional columns detailing clone library origin and sequence homology information. Click here for file

Additional file 2 **Selection of housekeeping genes for testis and brain tissue**. Further details of how qPCR analysis was carried out routinely and a discussion of how housekeeping genes were selected for testis and brain tissue. Also further details of the MCH1 and MCH2 qPCR analyses of individual brain samples are provided. Click here for file

Additional file 3 **Full version of Table 3 (Table 3s)**. Differentially expressed genes of the brain during precocious maturation. This version includes additional clones that were not definitively identified and includes two additional columns detailing clone library origin and sequence homology information. Click here for file
