# Supplementary material for: Testes and brain gene expression in precocious male and adult maturing Atlantic salmon (Salmo salar)
Source: BMC Genomics. 2010 Mar 30;11:211. doi: 10.1186/1471-2164-11-211 (PMC2996963; doi:10.1186/1471-2164-11-211)
Supplement: Additional file 1 — Full version of Table 1 (Table 1s). Differentially expressed genes of the testis during precocious maturation. This version includes additional clones that were not definitively identified and includes two additional columns detailing clone library origin and sequence homology information. [file 1471-2164-11-211-S1.DOC]

**Table 1s: Differentially expressed genes of the testis during precocious maturation.**

| **Library** | **^1^GenBank Accession No.** | **^4^Up or Down** | **Fold** | **Gene name** | **Length (% ID)** | **E-value** | **^2^Accession No. of Hit (Unigene if appropriate)** |
| --- | --- | --- | --- | --- | --- | --- | --- |
| Stim. HK leukocytes | GO944178 | **↓** | 2.0 | Alpha globin 1 | 437/437 (100%) | 0.0 | NM_001124550 |
| Stim. HK leukocytes | GO944180 | **↓** | 1.9 | Alpha-4 globin | 376/393 (95%) | 4E-178 | BT050340 |
| Maturing ovary | GO256596 | **↑** | 5.4 | Anti-leukoproteinase precursor/OVP-2 | 523/529 (98%) | 6E-112 | TC64926 |
| Maturing ovary | GO256601 | **↓** | 7.2 | Anti-mullerian hormone | 774/775 (99%) | 0 | NM_001123585 |
| Maturing ovary | GO256604 | **↑** | 16.4 | Apolipoprotein C-1 | 443/502 (88%) | 1E-153 | NM_001141362 |
| Trout liver confinement | CR943959 | **↑** | 5.7 | Apolipoprotein C-1 | 294/316 (93%) | 7E-129 | NM_001141362 |
| Trout liver confinement | CR944033 | **↑** | 14.9 | Apolipoprotein C-1 | 294/316 (93%) | 7E-129 | NM_001141362 |
| Maturing ovary | GO256602 | **↑** | 14.0 | Apolipoprotein C-1 | 285/287 (99%) | 2E-141 | NM_001141362 |
| Candidate | n/a | **↑** | 6.6 | Apolipoprotein E | n/a | n/a | CA050208 |
| Maturing female pituitary | GO256613 | **↑** | 2.9 | Apolipoprotein E | 573/573 (100%) | 0.0 | CB502338 (Ssa30932) |
| Smolt brain | GO256585 | **↓** | 2.6 | Beta globin | 450/469 (96%) | 0.0 | AY026061 |
| Stim. HK leukocytes | GO944177 | **↓** | 3.2 | Beta globin | 438/452 (97%) | 0.0 | YO8923 |
| Smolt head kidney | FD425634 | **↓** | 1.7 | Beta-2 microglobulin | 307/308 (99%) | 1E-64 | TC33956 |
| Maturing ovary | GO256603 | **↑** | 3.6 | Cathepsin B | 613/614 (99%) | 6E-134 | TC55653 |
| Maturing ovary | GO256605 | ** ↑** | 2.7 | Cathepsin B | 613/614 (99%) | 6E-134 | TC55653 |
| Smolt head kidney | FD425625 | **↓** | 3.7 | Collagen 1A2 | 169/170 (99%) | 6E-33 | TC37297 |
| Smolt head kidney | FD425605 | **↓** | 3.7 | Collagen 1A2 | 477/509 (93%) | 9E-100 | TC22424 |
| Smolt head kidney | FD425653 | **↓** | 4.5 | Collagen 1A2 | 477/509 (93%) | 9E-100 | TC22424 |
| Smolt brain | GO256584 | **↓** | 1.8 | Elongation factor EF-1 alpha | 461/470 (98%) | 0.0 | AF498320 |
| Candidate | n/a | **↓** | 1.8 | Elongation factor EF-1 alpha | n/a | n/a | CB502673  (Ssa.30552) |
| Full-length liver | BI468075 | **↓** | 2.4 | Elongation factor EF-1 gamma | 574/613 (93%) | 1E-121 | TC41574 |
| Candidate | n/a | **↓** | 1.5 | Elongation factor EF-2 | n/a | n/a | DV196880  (Omy.33489) |
| Smolt gill | FD425597 | **↑** | 2.9 | GDP-mannose 4, 6-dehydratase | 347/350 (99%) | 2E-73 | TC26943 |
| Smolt head kidney | FD425627 | **↑** | 1.1 | Glutamine synthetase | 214/214 (100%) | 3E-44 | TC28369 |
| Hypothalamus precocity | FD425678 | **↓** | 1.23 | Glutathione S-transferase | 493/523 (94%) | 6E-98 | TC117527 |
| Stim. HK leukocytes | GO944125 | **↓** | 2.1 | Glutathione S-transferase | 550/566 (97%) | 8E-116 | TC111174 |
| Trout pituitary confinement | GT222009 | **↓** | 1.6 | Growth hormone 1 precursor | 401/410(98%) | 0.0 | CX719563 |
| Smolt brain | GO256590 | **↓** | 2.6 | Guanine nucleotide binding protein/RACK1 | 451/456 (98%) | 0.0 | BT043532 |
| Trout pituitary confinement | GT222008 | **↑** | 1.6 | Heat shock protein hsp70a | 582/583 (99%) | 0.0 | NM_001124232 |
| Candidate | n/a | **↓** | 1.7 | Heat shock protein hsp90 beta | n/a | n/a | AJ632154  (Ssa.1060) |
| Full-length liver | BI468080 | **↓** | 1.4 | Heat shock protein hsp90 beta | n/a | n/a | BI468080. |
| Mature female hypothalamus | FD425670 | **↓** | 1.6 | Heat shock protein hsp90 beta | 147/161 (91%) | 4E-24 | TC22349 |
| Smolt gill | FD425588 | **↓** | 2.3 | Heat shock protein hsp90 beta | 744/752 (99%) | 0 | AF135117 |
| Candidate | n/a | **↑** | 1.9 | Gonadotropin-releasing hormone receptor | n/a | n/a | CA046044  (Omy.8048) |
| Testis precocity | GO256593 | **↑** | 2.7 | Lipoprotein lipase | 363/366 (99%) | 8E-77 | TC52451 |
| Maturing ovary | GO256597 | **↑** | 2.1 | Nuclear Protein-1 | 492/500 (98%) | 0 | BT048359 |
| Maturing female pituitary | GO256611 | **↑** | 2.6 | Proopiomelanocortine B | 172/173 (99%) | 8E-81 | DQ508935 |
| Smolt intestine | GT145199 | **↑** | 2.3 | Retinoic acid receptor responder protein 3 | 499/503 (99%) | 0.0 | EG875900 (Ssa.836) |
| Trout pituitary confinement | n/a | **↑** | 2.0 | 16S Ribosomal rRNA | 793/802 | 0.0 | DQ864465 |
| Smolt brain | GT145267 | **↓** | 2.4 | Ribosomal protein L8 | 162/178 (91%) | 7E-59 | AY957563 |
| Hypothalamus: mature v parr (female) | FD425672 | **↓** | 2.4 | Ribosomal protein S5 | 234/257 (91%) | 4E-89 | AF543539 |
| Stim. HK leukocytes | GO944179 | **↓** | 1.7 | Similar to CD209 antigen-like protein D | 49/52 | 0.012 | BT048497 |
| Smolt brain | GO256582 | **↓** | 7.9 | Similar to collagen 1A3 | 323/464 (69%) | 2E-36 | TC151122 (O.mykiss) |
| Pituitary precocity | GO256609 | **↑** | 1.4 | Similar to influenza virus NS1A binding protein b | 102/137 (74%) | 7E-12 | BC066513 |
| Maturing female pituitary | GO256612 | **↑** | 1.3 | Similar to neural cell adhesion L1-like | 312/318 (98) | 8E-151 | TC82871 |
| Smolt gill | FD425595 | **↓** | 2.0 | Similar to ribosomal protein L5 | 268/268 (100%) | 9E-134 | BT046397 |
| Smolt pituitary | FE963911 | **↑** | 1.4 | Similar to SIX homeobox 6 | 310/382 (81%) | 1E-90 | DW628904 |
| Testis precocity | GO256594 | **↓** | 6.0 | Transferrin | 302/302 (100%) | 3E-152 | BT045182 |
| Smolt brain | FE963864 | **↓** | 5.2 | Zinc finger protein Zic1 | 290/292 (99%) | 5E-143 | NM_001140488 |
| Smolt brain | GO256587 | **↓** | 2.4 | Common repetitive sequence | 294/294 (100%) | 2E-163 | EU025708 |
| Smolt hypothalamus | FD425661 | **↑** | 1.3 | Unknown EST | 133/161 (83%) | 3E-19 | BX081412 |
| Post-blastula transition | GO256620 | **↑** | 1.2 | Unknown EST | 518/521 (99%) | 0.0 | EG764643 |
| Post-blastula transition | GO256618 | **↑** | 1.5 | Unknown EST | 283/283 (100%) | 8E-157 | EG908436 |
| Mature female hypothalamus | GO256621 | **↑** | 1.4 | Unknown (short) EST | 46/46 (100%) | 1E-15 | GE470653 |
| Smolt head kidney | FD425628 | **↑** | 1.2 | Unknown (short) EST | 30/30 (100%) | n/a | n/a |
| Smolt head kidney | FD425631 | **↓** | 1.9 | Unknown (short) EST | 30/30 (100%) | n/a | n/a |
| Smolt brain | GO256583 | **↓** | 2.7 | Unknown EST | 458/477 (96%) | 4E-177 | CX261903 |
| Smolt brain | GO256591 | **↑** | 2.3 | Unknown EST | 86/97 & 100/117 | 5E-20 | CB513820 |
| Brain precocity | FD425567 | **↑** | 2.6 | Unknown EST | 57/60(95%) | 6E-17 | CK990366 |
| Trout brain confinement | GT222011 | **↑** | 1.7 | Unknown EST | 135/135 (100%) | 2E-70 | EG760376 |
| Full-length liver | n/a | **↓** | 2.9 | Unsequenced | n/a | n/a | n/a |
| Smolt head kidney | n/a | **↓** | 1.9 | Failed | n/a | n/a | n/a |
| Smolt brain | n/a | **↑** | 7.4 | Failed | n/a | n/a | n/a |
| Smolt brain | n/a | **↓** | 1.6 | Failed | n/a | n/a | n/a |
| Trout gonad | n/a | **↓** | 2.5 | Failed | n/a | n/a | n/a |
| Trout gonad | n/a | **↓** | 1.9 | Failed | n/a | n/a | n/a |

^1^Clones on this list may already have been sequenced in other projects (e.g. STRESSGENES and SALGENE). Accession numbers are given where available.

^2^BLAST accession numbers starting with TC indicate sequences in the TIGR database.

^3^Failed sequenced either gave very poor sequence or sequences that were too short for identification

^4^An upward arrow indicates up-regulation in precocious males
