# Supplementary material for: Testes and brain gene expression in precocious male and adult maturing Atlantic salmon (Salmo salar)
Source: BMC Genomics. 2010 Mar 30;11:211. doi: 10.1186/1471-2164-11-211 (PMC2996963; doi:10.1186/1471-2164-11-211)
Supplement: Additional file 2 — Selection of housekeeping genes for testis and brain tissue. Further details of how qPCR analysis was carried out routinely and a discussion of how housekeeping genes were selected for testis and brain tissue. Also further details of the MCH1 and MCH2 qPCR analyses of individual brain samples are provided. [file 1471-2164-11-211-S2.DOC]

Additional file 2

Title: Selection of housekeeping genes for testis and brain tissue

Description: Further details of how qPCR analysis was carried out routinely and a discussion of how housekeeping genes were selected for testis and brain tissue. Also further details of the MCH1 and MCH2 qPCR analyses of individual brain samples are provided.

Figure 1s: Comparison of Ct values for several housekeeping genes in testes samples

Figure 2s: Comparison of Ct values for several housekeeping genes in ovarian samples

Figure 3s: Comparison of Ct values for MCH1 and ubiquitin in precocious and non-precocious brain samples

Figure 4s: Relative expression of MCH1 and MCH2 in precocious and non-precocious brain samples

**Details on Quantitative Real-time PCR**

**Testes qPCR:**

The choice of housekeeping gene for gonads is problematic because the gonad has some tissue-specific forms of genes (e.g. GAPDH) and because the gonad is extremely physiologically active in some of the developmental stages being examined. Several potential housekeeping genes were analysed for use as reference genes in quantitative PCR. In order to identify a gene that could be reliably used as a reference in the precocious testes samples, in adult maturing testes samples and in adult mature/maturing ovarian samples in a linked study (Guiry and Cairns, unpublished) we examined expression of beta-actin (ACTB), glyceraldehyde-3-phosphate dehydrogenase (GAPDH), hypoxanthine-guanine phosphoribosyl transferase (HPRT), cyclophilin (CYC), ubiquitin (UBQ) and succinate dehydrogenase (SD). Primers were designed using VectorNTI Advance™software (Invitrogen) and size of amplicons ranged from 100 to 150 base pairs.

Pooled samples of precocious testes cDNA, non-precocious testes cDNA, July returning adult testes cDNA, November returning adult testes cDNA, multi-sea-winter (MSW) returning adult ovarian cDNA, July returning adult ovarian cDNA, September returning adult ovarian cDNA, October returning adult ovarian cDNA and November returning adult ovarian cDNA were used for this analysis. From a comparison of Ct values it was clear that ACTB, CYC and GAPDH varied significantly in both ovarian and testes samples, and that UBQ varied in testes samples. Of the 2 remaining genes (SD and HPRT) only SD showed no significant variation between the different testes samples or between the different ovarian samples, although like all the genes, ‘MSW’ ovaries (from July) and ‘July’ ovaries (one-sea-winter grilse) were quite different. All Ct values were therefore normalised to SD only.


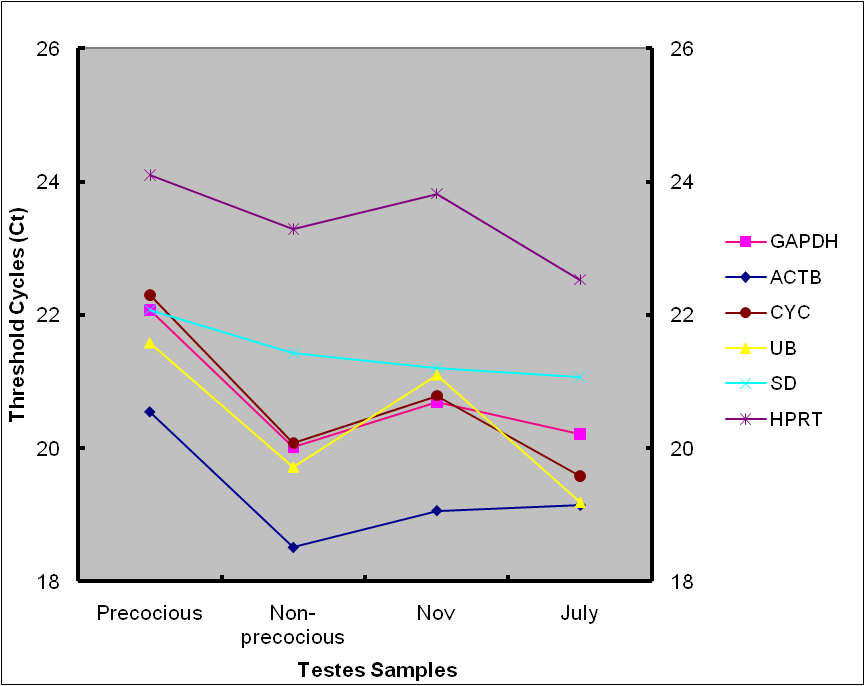


Figure 1s: Comparison of Ct values for several housekeeping genes in testes samples

Samples were analysed in triplicate and raw Ct values are presented for a number of housekeeping genes in testes samples. Nov, returning male grilse captured in November; July, returning male grilse captured in July.


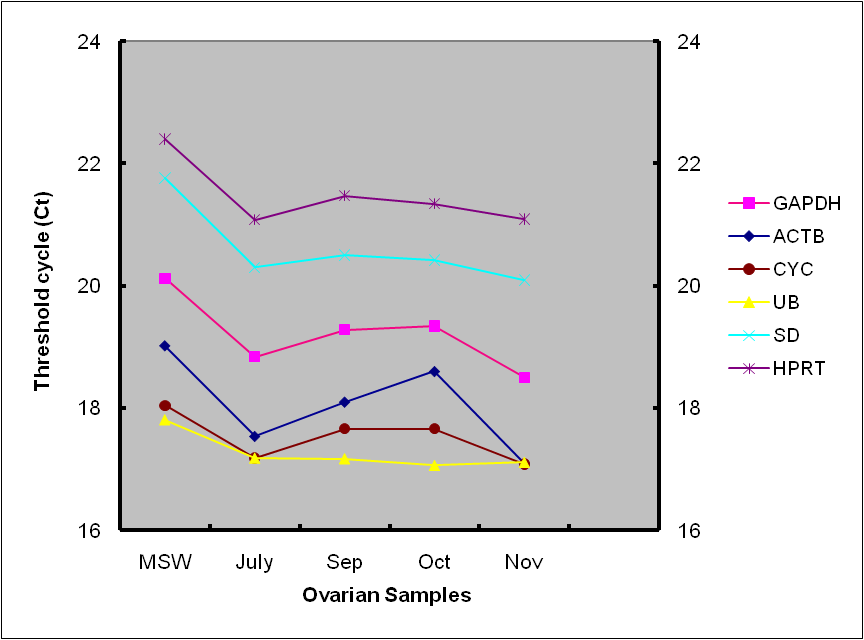


Figure 2s: Comparison of Ct values for several housekeeping genes in ovarian samples

Samples were analysed in triplicate and raw Ct values are presented for a number of housekeeping genes in ovarian samples. All are returning female grilse captured in July, September, October or November, except for ‘MSW’ which are returning multi-sea-winter females captured in July.

Routinely fresh cDNA (~10ng/μl) was diluted 1 in 10 and 1 in 100 and amplification was carried out in triplicate with neat, 1 in 10 and 1 in 100 cDNA to check for a linear response of signal to cDNA. Amplification efficiency was extracted from graphs but we rarely use any value other than 2 as, after optimising the reaction, we seldom see efficiencies corresponding to a cycle fold change of less than 1.95. Amplicons were checked for single products on ethidium bromide-stained agarose gels during optimisation, as were melting curves at the end of amplification.

Initially qPCR was carried out for 9 genes on the same pools used for microarray hybridisations (Fig. 4a) because this was a direct validation of the microarray analysis. Subsequently 4 genes were analysed in individuals (8 or 9 precocious males, 3 returning July males, 3 returning November males) or 4 non-precocious pools (each of 10 testes) to examine individual variation (Fig. 4b). (Non-precocious testes were only collected in pools of 10 as they were small: we therefore had to use 4 pools as a best possible alternative to individuals).

Considering the very large fold down-regulation changes in AMH in this study we do not presume that these changes are quantitatively reliable though relative to the different groupings (precocious v. non-precocious, etc) we consider that they give an accurate representation of the different levels of AMH. The lower limit (more dilute) of the efficiency curve could have been extended, a much more weakly expressed housekeeping gene could have been used (almost certainly impractical) or a diluted stock of cDNA (100-fold) could have been used for the housekeeper amplification.

**Brain qPCR:**

The approach taken for the brain samples was much simpler. Previous studies in our laboratory on rainbow trout (another salmonid) had routinely shown that ubiquitin was a very stably expressed gene in brain tissue (as too was beta-actin in our experience). Many papers in the literature have shown that ubiquitin is a robust brain housekeeping gene. Primers were designed for qRT-PCR using VectorNTI Advance™software (Invitrogen). Size of the amplicons ranged from 100 to 150 base pairs.

For brain qPCR most of the comparisons between precocious and non-precocious fish were made using pools (either PB1 or PB2 against NPB2). We found no significant difference in UBQ levels between the groups. However, we did look at MCH1 and MCH2 in 5 individual fish of each group (PB2 and NPB2). The Ct values for the non-precocious samples do show signs of being lower especially in samples NPB9 and NPB10 (Fig. 3S). On average MCH1 was up-regulated approximately 1.7 fold in precocious males. There was considerable variability in the levels of MCH1 in the ten individuals (Fig. 4S). An approximate 290-fold difference between the highest and the lowest individuals may reflect sampling inconsistency as to the specific parts of the brain included. This reduces to 120-fold if straight Ct values are subtracted, i.e. without normalisation which might be in question since the Ct value for the reference gene (SD) is abnormally low for NPB10. This seems an exception level of variation in a brain gene but is supported by the MCH2 levels which approximately follow the MCH1 trend across individuals (before correction). MCH2 levels (C_t_~34) were at least 100 times lower than MCH1 levels (C_t_~26) so comparisons of MCH2 levels between individuals were not reliable.


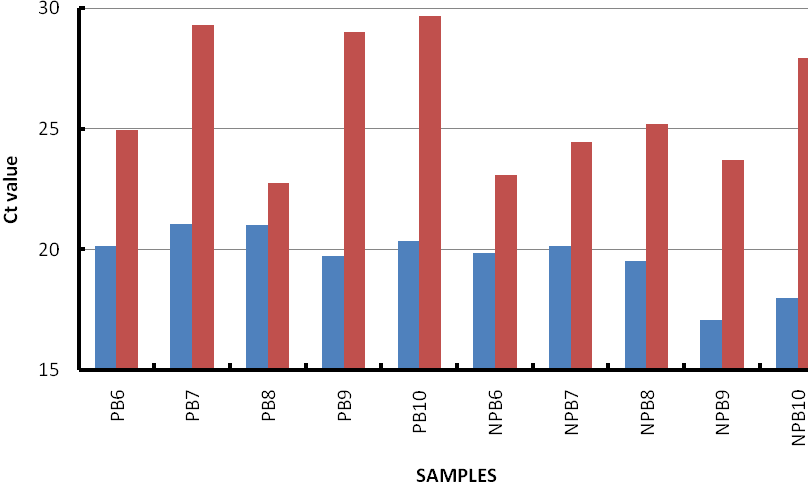


Figure 3s: Comparison of Ct values for MCH1 and ubiquitin in precocious and non-precocious brain samples

Samples were analysed in triplicate and raw Ct values are presented. The housekeeping gene ubiquitin (UBQ) is shown in blue bars and MCH1 in red bars. PB6-PB10 refers to precocious brain samples and NPB6-NPB10 refers to non-precocious brain samples each from 5 individual fish.


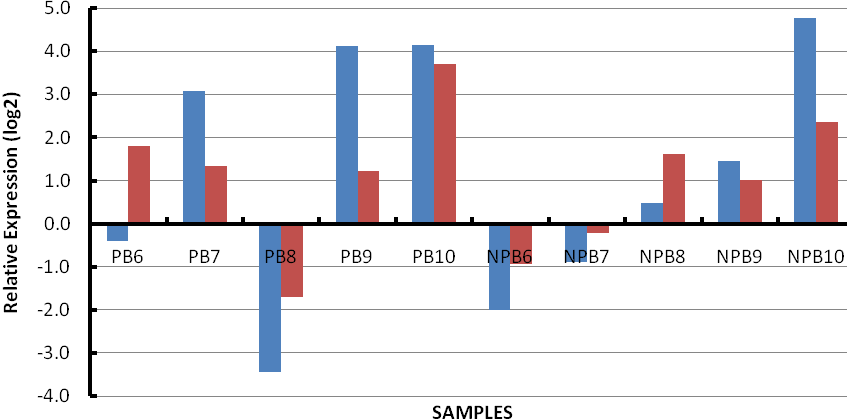


Figure 4S: Relative expression of MCH1 and MCH2 in precocious and non-precocious brain samples

Samples were analysed in triplicate and relative expression levels (normalised to the housekeeping gene ubiquitin) are given as log2 values. MCH1 is shown in blue bars and MCH2 in red bars. PB6-PB10 refers to precocious brain samples and NPB6-NPB10 refers to non-precocious brain samples each from 5 individual fish.
