# Supplementary material for: Testes and brain gene expression in precocious male and adult maturing Atlantic salmon (Salmo salar)
Source: BMC Genomics. 2010 Mar 30;11:211. doi: 10.1186/1471-2164-11-211 (PMC2996963; doi:10.1186/1471-2164-11-211)
Supplement: Additional file 3 — Full version of Table 3 (Table 3s). Differentially expressed genes of the brain during precocious maturation. This version includes additional clones that were not definitively identified and includes two additional columns detailing clone library origin and sequence homology information. [file 1471-2164-11-211-S3.DOC]

**Table 3s:** **Differentially expressed genes of the brain during precocious maturation.**

| **Library** | **^1^GenBank Accession No.** | **Up or Down** | **Fold** | **Gene name** | **Length (% ID)** | **E-value** | **^2^Accession No. of Hit (Unigene if appropriate)** |
| --- | --- | --- | --- | --- | --- | --- | --- |
| Full-length spleen | BE518469 | **↓** | 1.5 | Alpha-globin 1 | 492/515 (95%) | 0.0 | NM_001124550 |
| Stim. HK leukocytes | GO944156 | **↓** | 1.3 | Alpha-globin 1 | 121/123 (98%) | 0.0 | NM_001124550 |
| Full-length spleen | BE518469 | **↓** | 1.3 | Alpha-globin 1 | 492/515 (95%) | 0.0 | NM_001124550 |
| Trout gonad | GR209362 | **↓** | 1.4 | Alpha-globin 4 | 210/211 (99%) | 5E-101 | BT050340 |
| Stim. HK leukocytes | GO944180 | **↓** | 1.5 | Alpha-globin 4 | 376/393 (95%) | 4E-178 | BT050340 |
| Smolt pituitary | GT145313 | **↓** | 1.2 | Anamorsin | 468/468 (100%) | 0.0 | BT043538 |
| Candidate | n/a | **↓** | 1.3 | Apolipoprotein E | n/a | n/a | CA050208 Ssa.30932 |
| Mature female adult v grilse pituitary | GO256613 | **↓** | 1.3 | Apolipoprotein E | 566/566 (100%) | 2E-123 | TC91898 |
| Full-length spleen | BE518484 | **↓** | 1.3 | Beta-2 microglobulin | 409/411 (99%) | 0.0 | NM_001123699 |
| Candidate | n/a | **↓** | 1.2 | Beta-actin | n/a | n/a | NM_001123525 |
| Smolt pituitary | GO256610 | **↑** | 1.2 | Beta-catenin interacting protein 1 | 531/532 (99%) | 0.0 | NM_001139923 |
| Smolt brain | GO256585 | **↓** | 1.5 | Beta globin | 450/469 (96%) | 0.0 | AY026061 |
| Stim. HK leukocytes | GO944177 | **↓** | 1.4 | Beta globin | 438/452 (97%) | 0.0 | YO8923 |
| Full-length spleen | BE518474 | **↓** | 1.5 | Beta-globin | 599/599 (100%) | 0.0 | NM_001123666 |
| Candidate | n/a | **↓** | 1.2 | Beta-tubulin | n/a | n/a | NM_001139793 |
| Smolt hypothalamus | GO256580 | **↓** | 1.3 | Beta-tubulin | 268/305 (87%) | 2E-103 | NM_001140292 |
| Trout brain confinement | CR943970 | **↓** | 1.3 | Beta tubilin | 129/134 (96%) | 8E-54 | BT058720 |
| Candidate | n/a | **↓** | 1.3 | Cathepsin D | n/a | n/a | NM_001124711 |
| Smolt brain | FE963874 | **↓** | 1.2 | Cofilin 2 | 178/178 (100%) | 2E-94 | BT060221 |
| Candidate | n/a | **↓** | 1.2 | Elongation factor EF-1 alpha | n/a | n/a | CB502673 |
| Smolt brain | GO256584 | **↓** | 1.2 | Elongation factor EF-1 alpha | 275/275 (100%) | 4E-152 | BT058929 |
| Smolt brain | GO256588 | **↓** | 1.2 | Elongation factor EF-1 alpha | 444/470 (94%) | 0.0 | NM_001141909 |
| Stim. HK leukocytes | GO944131 | **↓** | 1.1 | Ferritin H-1 | 223/227 (98%) | 3E-112 | NM_001124547 |
| Candidate | n/a | **↓** | 1.2 | GAPDH | n/a | n/a | NM_001123561 |
| Candidate | n/a | **↓** | 1.2 | GAPDH | n/a | n/a | NM_001123561 |
| Smolt pituitary | n/a | **↓** | 1.2 | Glutamine synthetase | 70/93 (75%) | 3E-10 | NM_001124314 |
| Smolt brain | GO256590 | **↓** | 1.2 | Guanine nucleotide binding protein, beta polypeptide 2-like 1 | 451/456 (98%) | 0.0 | BT059305 |
| Trout pituitary confinement | GT222010 | **↑** | 1.2 | Heterogeneous nuclear ribonucleoprotein A/B | 392/432 (90%) | 2E-106 | BT045277 |
| Candidate | n/a | **↑** | 1.2 | Hydroxysteroid 11-beta dehydrogenase | n/a | n/a | AB104415 |
| Full length spleen | BE518475 | **↑** | 1.2 | Interleukin enhancer binding factor 2 | 163/171 (95%) | 1E-70 | BT045275 |
| Candidate | n/a | **↓** | 1.5 | Isotocin 2 | n/a | n/a | NM_001123652 |
| Candidate | n/a | **↑** | 1.2 | Melanin-concentrating hormone 2 | n/a | n/a | M25755 |
| Smolt brain | GT145268 | **↓** | 1.2 | MHC class I | 742/756 (98%) | 0.0 | AF504013 |
| Smolt spleen | GT145243 | **↓** | 1.2 | MHC class I | 686/689 (99%) | 0.0 | AF504016 |
| Smolt brain | FE963873 | **↓** | 1.2 | ^4^Myelin P0-like glycoprotein | 227/251 (90%) | 7E-90 | NM_001140077 |
| Trout gonad | GR209337 | **↓** | 1.2 | Myosin-9 (putative) | 523/574 (91%) | 0.0 | BT072044 |
| Stim. HK leukocytes | GO944099 | **↑** | 1.2 | Phosphogluconate dehydrogenase | 190/194 (97%) | 2E-94 | BT059099 |
| Trout brain confinement | CR944012 | **↓** | 1.3 | Phosphoglycerate mutase 1 | 140/141 (99%) | 2E-64 | CX249369 (Omy.36005) |
| Smolt hypothalamus | GO256580 | **↑** | 1.2 | Proline-rich nuclear receptor coactivator 2 | 532/538 (98%) | 0.0 | NM_001140247 |
| Trout liver confinement | CR944197 | **↑** | 1.3 | Reverse transcriptase-like protein | 408/408 (100%) | 0.0 | CR944197 |
| Full length testis | BM413842 | **↓** | 1.2 | 60S Ribosomal protein L13A | 476/476 (100%) | 0.0 | BT044039 |
| Trout brain confinement | CR943861 | **↓** | 1.2 | S100 calcium binding protein | 604/663 (91%0 | 0.0 | NM_001146376 |
| Full length testis | BM413745 | **↓** | 1.2 | Simple type II keratin K8 | 563/620 (90%) | 0.0 | NM_001124734 |
| Full length brain | BF228584 | **↑** | 1.4 | Suppressor of G2 allele of SKP1 | 505/505 (100%) | 0.0 | BT071868 |
| Full length brain | BF228584 | **↓** | 1.3 | Suppressor of G2 allele of SKP1 | 505/505 (100%) | 0.0 | BT071868 |
| Candidate | n/a | **↑** | 1.2 | TGFB2 | n/a | n/a | Aj318936 |
| Smolt brain | GO256586 | **↓** | 1.2 | VAMP-2 | 347/347 (100%) | 2E-176 | CA060527 Ssa.21646 |
| Candidate | n/a | **↓** | 1.7 | Vasotocin-1 | n/a | n/a | DY736376 |
| Candidate | n/a | **↓** | 1.9 | Vasotocin-1 | n/a | n/a | DY736376 |
| Trout brain confinement | CR943901 | **↑** | 1.2 | Common repetitive sequence | n/a | n/a | n/a |
| Mature female hypothalamus | GO256622 | **↓** | 1.2 | Common repetitive sequence | n/a | n/a | n/a |
| Hypothalamus precocity | GO256615 | **↓** | 1.3 | Common repetitive sequence | 349/459 (76%) | 7E-93 | EG776437 |
| Smolt brain | FE963863 | **↑** | 1.7 | Common repetitive sequence | n/a | n/a | n/a |
| Smolt pituitary | FE963931 | **↓** | 1.5 | Unknown EST (brain) | 509/516 (98%) | 0.0 | CB510712 |
| Smolt brain | GO256589 | **↓** | 1.3 | Unknown EST (brain) | 332/358 (89%) | 3E-141 | DW551846 |
| Brain precocity | FD425534 | **↓** | 1.3 | Unknown EST (brain) | 561/570 (98%) | 0.0 | EG771556 (Ssa.6062) |
| Mid blastula transition | GO256620 | **↑** | 1.2 | Unknown EST | 453/460 (98%) | 0.0 | EG769489 |
| Smolt brain | GO256592 | **↑** | 1.4 | Unknown EST | 397/397 (100%) | 0.0 | GE783707 |
| Smolt pituitary | FE963938 | **↑** | 1.1 | Unknown EST (very short) | n/a | n/a | n/a |
| Hypothalamus precocity | GO256617 | **↓** | 1.3 | Unknown EST | 108/125 (86%) | n/a | EG766468 |
| Hypothalamus precocity | FD425679 | **↑** | 1.3 | Unknown EST (common repetitive element) | 160/160 (100%) | 5E-75 | EG764477 |
| Smolt gill | GO256627 | **↓** | 1.2 | Unknown EST | 415/415 (100%) | 0.0 | EG841454 |
| Smolt gill | GO256624 | **↓** | 1.2 | Unknown EST | 335/339 (98%) | 5E-167 | EG841454 |
| Smolt pituitary | FE963942 | **↑** | 1.1 | Unknown EST | 316/316 (100%) | 3E-176 | DW544916 |
| Smolt gill | GO256626 | **↓** | 1.2 | Unknown DNA | n/a | n/a | n/a |
| Trout gonad | GT222007 | **↓** | 1.2 | Unknown DNA | 417/476 (87%) | 4E-167 | DY728572 |

See footnotes to Table 1. In addition:

^4^First 539bp do not align to myelin P0-like glycoprotein (S.salar): 318/321 (99%) align to S. salar EST GE774561.
